# Dual action of L-Lactate on the activity of NR2B-containing NMDA receptors: from potentiation to neuroprotection

**DOI:** 10.1038/s41598-018-31534-y

**Published:** 2018-09-07

**Authors:** P. Jourdain, K. Rothenfusser, C. Ben-Adiba, I. Allaman, P. Marquet, P. J. Magistretti

**Affiliations:** 1 Brain Mind Institute, Ecole Polytechnique Fédérale de Lausanne (EPFL), 1015 Lausanne, Switzerland; 2 Unité Mixte Internationale, Université de Lausanne-Université Laval, Département de Psychiatrie-CHUV, CH-1008 Prilly/Lausanne, Switzerland; 3 CERVO Brain Research Centre, Québec Mental Health Institute, Quebec City, QC Canada; 4 Center for Optics, Photonics and Lasers (COPL), Laval University, Quebec City, QC Canada; 5 Department of Psychiatry and Neuroscience, Université Laval, Québec City, QC Canada; 6 King Abdullah University of Science and Technology (KAUST), Thuwal, Saudi Arabia

## Abstract

L-Lactate is a positive modulator of NMDAR-mediated signaling resulting in plasticity gene induction and memory consolidation. However, L-Lactate is also able to protect neurons against excito-toxic NMDAR activity, an indication of a mitigating action of L-Lactate on NMDA signaling. In this study, we provide experimental evidence that resolves this apparent paradox. Transient co-application of glutamate/glycine (1 μM/100 μM; 2 min) in primary cultures of mouse cortical neurons triggers a NMDA-dependent Ca^2+^ signal positively modulated by L-Lactate (10 mM) or DTT (1 mM) but decreased by Pyruvate (10 mM). This L-Lactate and DTT-induced potentiation is blocked by Ifenprodil (2 μM), a specific blocker of NMDARs containing NR2B sub-units. In contrast, co-application of glutamate/glycine (1 mM/100 μM; 2 min) elicits a NMDAR-dependent excitotoxic death in 49% of neurons. L-Lactate and Pyruvate significantly reduce this rate of cell death processes (respectively to 23% and 9%) while DTT has no effect (54% of neuronal death). This L-Lactate-induced neuroprotection is blocked by carbenoxolone and glibenclamide, respectively blockers of pannexins and K_ATP_. In conclusion, our results show that L-Lactate is involved in two distinct and independent pathways defined as NMDAR-mediated potentiation pathway (or NADH pathway) and a neuroprotective pathway (or Pyruvate/ATP pathway), the prevalence of each one depending on the strength of the glutamatergic stimulus.

## Introduction

NMDA receptors (NMDARs) are glutamate-gated cation channels with high calcium permeability. They are involved in several aspects of brain activity such as neuronal development and neuroplasticity during learning and memory formation^1^. However, an overstimulation of NMDARs leads to neuronal damage (excitotoxicity), as illustrated by a variety of neurological disorders and pathological conditions^2^ such as ischemic brain injury and chronic neurodegenerative diseases such as Alzheimer’s disease or Amyotrophic Lateral Sclerosis^3,4^. NMDARs require two distinct molecules to be active: glutamate as the main agonist and a co-agonist, either glycine or D-Serine. Other endogenous substances found in the central nervous system can also act as potent modulators of NMDAR activity, for example protons, Zn^2+^, polyamines^5^ or L-Lactate^6^. The latter molecule has long been considered as a waste product of Glucose metabolism with no specific functions. This view of L-Lactate has however changed during the past three decades from waste product to fuel for neurons through the concept of the Astrocyte-Neuron Lactate Shuttle^7,8^ where astrocyte-derived L-Lactate acts as an energy substrate to meet the increased energy demands of neurons during neuronal activity. Furthermore, in addition to its role in energy metabolism, L-Lactate also acts as a signaling molecule for neuronal plasticity and Long-Term Memory formation^9,10^ and for neuroprotection^11–14^. These two sets of properties of L-Lactate, namely neuroplasticity and neuroprotection (for review see^15^), may appear at first analysis contradictory. Indeed the positive modulation of NMDARs by L-Lactate^6^ associated with neuroplasticity seems hard to reconcile with the neuroprotective action of L-Lactate against NMDAR-mediated excito-toxicity^14^. This apparent discrepancy is particularly reinforced by the fact that the positive modulation of NMDAR activity by L-Lactate is associated with an increase in the redox potential of neurons as determined by the NADH/NAD^+^ ratio^6^. Such redox conditions are known to increase the activity of NMDARs^16,17^ to a point where they may cause glutamate-evoked neurotoxicity^18^.

To address this apparent paradox, we performed a series of Ca^2+^ imaging studies using Fura-2 to measure [Ca^2+^]_i_ elevations in cultured neurons before, during and after glutamate stimulation at concentrations of glutamate ranging from 1 μM to 1 mM. Results reported in this article demonstrate that L-Lactate exerts two distinct effects on neuronal [Ca^2+^]_i_ depending on the concentration of glutamate: potentiation of low (1 μM, subthreshold) and mitigation of elevated (1 mM, excito-toxic) concentrations of glutamate respectively. The potentiation by L-Lactate on NMDAR signaling in the presence of low glutamate concentrations involves the formation of NADH and modulation of the NR2B subunit. The mitigation of the [Ca^2+^]_i_ increases elicited by excito-toxic concentrations of glutamate, are shared by Pyruvate and involve the formation of ATP, consistent with the previously described neuroprotective effect of L-Lactate and Pyruvate^14^.

## Results

In this study, all Ca^2+^-responses induced by different concentrations of glutamate co-applied with a fixed and saturating concentration of glycine (100 μM) are recorded from cultured neurons aged to 17–22 DIV.

### Co-application of glutamate and glycine evokes a Ca^2+^ signal associated with activation of NMDARs through a NR2B independent pathway

**Figure 1:**
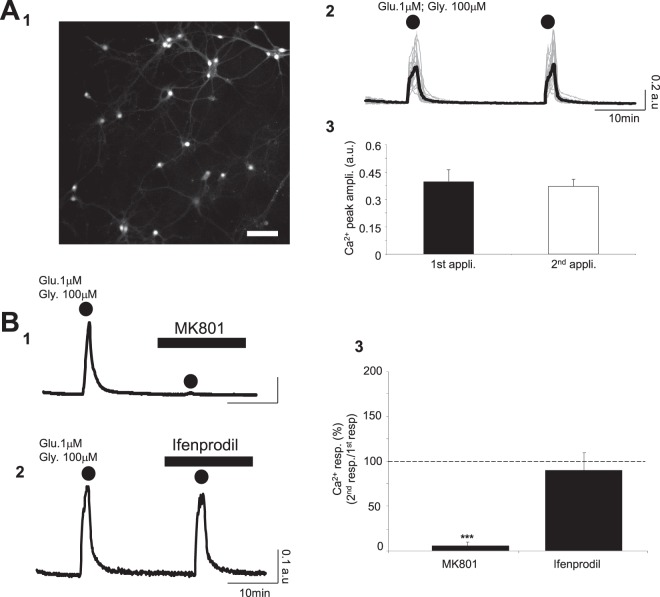
Co-application of glutamate and glycine evokes a Ca^2+^ signal associated with activation of NMDARs through an NR2B independent pathway. (**A**_**1–3**_) Typical fluorescence images of cortical neurons labelled with Fura-2 (380 nm excitation) (Scale Bar: 100 μm; **A**_**1**_). Intracellular calcium signals (grey – 27 individual and black – average calcium traces recorded from cultured neurons in **A**_**1**_) following 2 successive co-applications of glutamate and glycine (2 min; dots; **A**_**2**_). Calcium traces indicate that there is no detectable difference between the 2 successive Ca^2+^ responses evoked by the co-application of glutamate/glycine. Bar charts comparing the amplitude for Ca^2+^ responses evoked by 2 successive co-application of glutamate/glycine (n_cult_ = 9; n_cells_ = 275; **A**_**3**_). (**B**_**1–3**_) Intracellular Ca^2+^ signals (black–average calcium traces recorded from 19 neurons in **B**_**1**_ and 37 neurons in **B**_**2**_) following 2 successive applications of glutamate/glycine (2 min; dots) in control condition (1^st^ application) and in presence of the broad-spectrum NMDAR antagonist MK801 (40 μM; **B**_**1**_) or a specific blocker of the NR2B sub-unit Ifenprodil (2 μM; **B**_**2**_). The 2^nd^ Ca^2+^ response induced by the co-application of glutamate/glycine is totally blocked by MK801 (**B**_**1**_) but not by Ifenprodil (**B**_**2**_). Bar chart summarizing the significant inhibitory effect of MK801 (n_cult_ = 8; n_cells_ = 271) and the lack of effect of Ifenprodil (n_cult_ = 7; n_cells_ = 299) on Ca^2+^ responses evoked by the co-application of glutamate/glycine (**B**_**3**_). Results are presented as means ± SEM (***p < 0.005; paired *t*-test).

NMDARs are highly permeable to Ca^2+^; their activation requires the simultaneous presence glutamate and glycine^19,20^. In a first series of experiments, we applied a low concentration of glutamate (1 μM) corresponding to the resting concentration of glutamate in extracellular space^21^ in conjunction with glycine (100 μM). As shown in Fig. 1A, co-application of glutamate/glycine during 2 min triggers a Ca^2+^ signal with a mean amplitude of 0.39 ± 0.07 a.u. (n_cult_ = 9; n_cells_ = 275). When the same glutamate/glycine cocktail is applied to the same cultures after a minimum delay of 20 min, a second Ca^2+^ response of 0.36 ± 0.06 a.u. is observed, an amplitude which is not significantly different from the first one (p > 0.05; Fig. 1A and Table 1). To confirm that this excitatory stimulus activates NMDARs, MK801, a specific antagonist of NMDARs was used. In the presence of MK801 (40 μM), the [Ca^2+^]_i_ elevation normally induced by the co-application of glutamate/glycine is totally blocked with a Ca^2+^ peak amplitude of 0.37 ± 0.06 a.u. in control condition *vs* 0.04 ± 0.01 a.u. with MK801 (p < 0.005; n_cult_ = 8; n_cells_ = 271) (Fig. 1B and Table 1).

**Table 1:** Summary of the Ca^2+^ peak amplitude induced by 2 successive applications of glutamate/glycine in different conditions.

Glutamate/Glycine (1 μM/100 μM; 2 min)	1^st^ application (control)	2^nd^ application (test)	% of effect	Paired *t-*Test (2^nd^ appl. *vs* 1^st^ appl.)
Control (n_cult_ = 9; n_cells_ = 275)	0.39 ± 0.07	0.36 ± 0.06	91.7 +−/ 14.8	*n*.*s*
MK801 (40 μM) (n_cult_ = 8; n_cells_ = 271)	0.37 ± 0.06	0.04 ± 0.01	9.4 ± 2.7	<0.005
Ifenprodil (20 μM) (n_cult_ = 7; n_cells_ = 299)	0.33 ± 0.07	0.28 ± 0.06	84.8 ± 17.3	*n*.*s*
L-Lactate (2 mM) (n_cult_ = 9; n_cells_ = 376)	0.32 ± 0.03	0.35 ± 0.04	110.2 ± 11.8	*n*.*s*
L-Lactate (5 mM) (n_cult_ = 10; n_cells_ = 365)	0.35 ± 0.06	0.34 ± 0.07	95.4 ± 14.8	*n*.*s*
L-Lactate (10 mM) (n_cult_ = 11; n_cells_ = 468)	0.33 ± 0.05	0.50 ± 0.05	152.8 ± 11.4	<0.005
Pyruvate (10 mM) (n_cult_ = 9; n_cells_ = 317)	0.37 ± 0.08	0.19 ± 0.09	51.6 ± 19.2	<0.05
D-Lactate (10 mM) (n_cult_ = 9; n_cells_ = 326)	0.37 ± 0.07	0.19 ± 0.07	50.7 ± 17.6	<0.05
5 mM extra Glucose (n_cult_ = 8; n_cells_ = 425)	0.29 ± 0.04	0.14 ± 0.06	47.2 ± 19.6	<0.05
L-Lactate (10 mM) + Ifenprodil (40 μM) (n_cult_ = 8; n_cells_ = 315)	0.35 ± 0.07	0.34 ± 0.04	96.4 ± 11.4	*n*.*s*
L-Lactate (with NMDA 30 μM + glycine 100 μM) (n_cult_ = 9; n_cells_ = 340)	0.34 ± 0.04	0.42 ± 0.04	122.6 ± 9.2	<0.05
L-Lactate (with D-Serine 100 μM) (n_cult_ = 9; n_cells_ = 317)	0.33 ± 0.07	0.45 ± 0.06	137.4 ± 18.9	*n*.*s*
DTT (1 mM) (n_cult_ = 9; n_cells_ = 343)	0.31 ± 0.04	0.52 ± 0.08	169.3 ± 23.2	<0.05
L-Lactate (10 mM) + DTNB (200 μM) (n_cult_ = 8; n_cells_ = 407)	0.34 ± 0.06	0.32 ± 0.06	92.7 ± 16.2	*n*.*s*
L-Lactate (10 mM) + Stiripentol (200 μM) (n_cult_ = 9; n_cells_ = 298)	0.31 ± 0.06	0.26 ± 0.05	82.5 ± 12.9	*n*.*s*
DTT (1 mM) + Ifenprodil (2 μM) (n_cult_ = 8; n_cells_ = 301)	0.37 ± 0.06	0.33 ± 0.05	89.4 ± 14.3	*n*.*s*
Lactate (10 mM) + Ryanodine (10 μM) (n_cult_ = 7; n_cells_ = 331)	0.32 ± 0.01	0.41 ± 0.03	129.6 ± 10.1	<0.05
**Glutamate/Glycine (10** **μM/100** **μM; 2** **min)**	**1** ^**st**^ **application (control)**	**2** ^**nd**^ **application (test)**	**% of effect**	**Paired** ***t-*Test (1**^**st**^ **appl**. ***vs*** **2**^**nd**^ **appl**.**)**
Ifenprodil (2 μM) (n_cult_ = 6; n_cells_ = 187)	0.52 ± 0.06	0.32 ± 0.06	61.3 ± 10.8	<0.005
L-Lactate (10 mM) (n_cult_ = 6; n_cells_ = 276)	0.49 ± 0.02	0.51 ± 0.03	103 ± 5.3	*n*.*s*
**Glutamate/Glycine (100** **μM/100** **μM; 2** **min)**	1^st^ application (control)	2^nd^ application (test)	% of effect	Paired *t-*Test (1^st^ appl. *vs* 2^nd^ appl.)
Ifenprodil (2 μM) (n_cult_ = 6; n_cells_ = 138)	0.59 ± 0.08	0.42 ± 0.09	71.4 ± 15.7	<0.005
L-Lactate (10 mM) (n_cult_ = 6; n_cells_ = 155)	0.55 ± 0.05	0.49 ± 0.07	88.2 ± 12	*n*.*s*

Values are means ± SEM. Statistics data correspond to a Paired Student’s *t*-test (*n*.*s*: non significant, p > 0.05).

NMDARs are a heteromeric complexes composed of the NR1 and at least one of the NR2 sub-units. The NR2 sub-units, in particular NR2A and NR2B, two sub-units expressed in primary cortical neuronal cultures, strongly influence the kinetics and pharmacological properties of NMDARs^22^. To differentiate the involvement of either NR2A or NR2B, we used Ifenprodil, a specific blocker of NR2B-containing NMDARs. Bath application of Ifenprodil (2 μM), did not affect the Ca^2+^ responses evoked by the glutamate/glycine application (control: 0.33 ± 0.06 a.u.; Ifenprodil: 0.28 ± 0.06 a.u.; n_cult_ = 7; n_cells_ = 299) (Fig. 1B and Table 1). This observation indicates that the glutamate/glycine-induced Ca^2+^ signals in control condition does not involve NR2B-containing NMDARs.

### L-Lactate specifically potentiates the Ca^2+^ signal evoked by the co-application of glutamate and glycine through activation of NR2B-containing NMDARs

**Figure 2:**
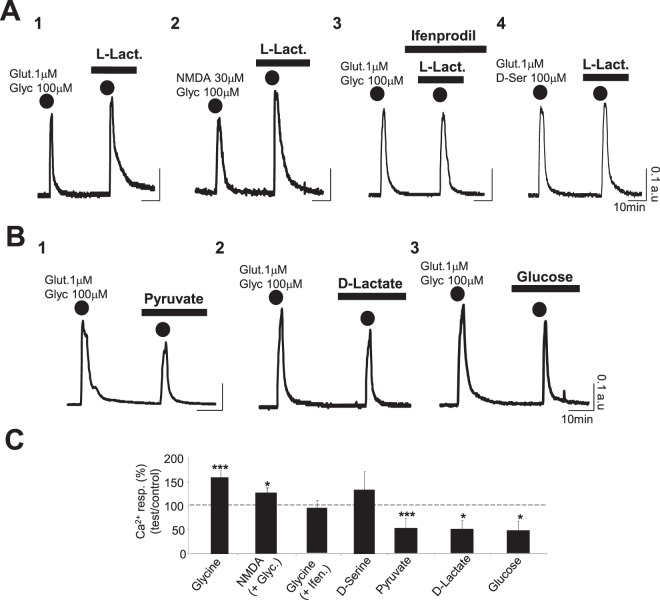
L-Lactate specifically potentiates the Ca^2+^ signal evoked by the glutamate/glycine cocktail through activation of NR2B-containing NMDARs: an effect associated to an increase of the intracellular NADH/NAD ratio. (**A**_**1–4**_) Intracellular Ca^2+^ signals (average Fura-2 ratio) recorded from 4 different cultures (**A**_**1**_: n_cell_ = 37; **A**_**2**_: n_cell_ = 35; **A**_**3**_: n_cell_ = 27; **A**_**4**_: n_cell_ = 50) and following 2 successive co-applications of glutamate/glycine (2 min; dots; **A**_**1**_ and **A**_**3**_), NMDA/glycine (2 min; dots; **A**_**2**_) and glutamate/D-Serine (2 min; dots; **A**_**3**_) in presence of L-Lactate alone (10 mM; **A**_**1**_,**A**_**2**_ and **A**_**4**_) or L-Lactate + Ifenprodil (2 μM; **A**_**3**_) for the 2^nd^ co-application. Regardless to the configuration, calcium traces clearly indicate that L-Lactate induces potentiation of the Ca^2+^ responses for the co-application of glutamate/glycine (**A**_**1**_) or NMDA/glycine (**A**_**2**_) this L-Lactate-induced potentiation being blocked by Ifenprodil (**A**_**3**_) and lacking when glycine is substituted by D-Serine (**A**_**4**_). (**B**_**1–3**_) Intracellular calcium signals (average Fura-2 ratio) following 2 successive co-applications of glutamate/glycine (2 min; dots) and recorded from 3 different cultures in presence of Pyruvate (10 mM) (n_cell_ = 20; **B**_**1**_), D-Lactate (10 mM) (n_cell_ = 55; **B**_**2**_) or Glucose (5 mM extra Glucose ACSF) (n_cell_ = 62; **B**_**2**_). The Ca^2+^ traces show clearly that neither Pyruvate (**B**_**1**_) nor D-Lactate (**B**_**2**_) are able to potentiate the Ca^2+^ response evoked by the co-application of glutamate/glycine. (**C**) Bar charts summarizing the significant potentiating effects of L-Lactate on Ca^2+^ responses evoked by the co-application of glutamate/glycine (n_cult_ = 11; n_cells_ = 468) or NMDA/glycine (n_cult_ = 9; n_cells_ = 343). In contrast, see the lack of L-Lactate-induced potentiation in presence of Ifenprodil (n_cult_ = 8; n_cells_ = 315) or when glycine is substituted by D-Serine (n_cult_ = 9; n_cells_ = 317). Finally, Pyruvate (n_cult_ = 9; n_cells_ = 317), D-Lactate (n_cult_ = 9; n_cells_ = 326) or Glucose (n_cult_ = 8; n_cells_ = 425) significantly decrease the Ca^2+^ signal. Results are presented as means ± SEM (*p < 0.05; ***p < 0.005; paired t-test).

In the presence of L-Lactate (10 mM), the Ca^2+^ response induced by glutamate/glycine (1 μM/100 μM; 2 min) is significantly increased with a Ca^2+^ peak amplitude rising from 0.33 ± 0.05 a.u. in control to 0.50 ± 0.05 a.u. in L-Lactate condition (p < 0.005; n_cult_ = 11; n_cells_ = 468) (Fig. 2A,C and Table 1). This effect depends upon NMDAR activation since direct NMDAR activation by the selective agonist NMDA (by co-application NMDA (30 μM) and Glycine (100 μM) during 2 min) is also significantly potentiated by L-Lactate (Ca^2+^ peak amplitude rising from 0.34 ± 0.04 a.u. to 0.42 ± 0.04; n_cult_ = 9; n_cells_ = 340, p < 0.05) (Fig. 2A,C and Table 1). Moreover, in the presence of Ifenprodil (2 μM), a selective blocker of the NR2B subunit of NMDARs, in the bath medium, L-Lactate (10 mM) no longer potentiates the Ca^2+^ response triggered by glutamate/glycine which is identical to the one obtained in control condition (Fig. 2A,C and Table 1). Finally, when glycine was substituted by D-serine, another endogenous co-agonist of the NMDARs, L-Lactate also slightly increased the Ca^2+^ signal evoked by the cocktail glutamate/D-serine (1 μM/100 μM; 2 min) from 0.33 +/0.07 a.u. to 0.45 ± 0.06 a.u. (n_cult_ = 9; n_cells_ = 317); this increase was however not statistically significant (Fig. 2A,C and Table 1). Since D-serine preferentially acts on NR2A-containing NMDARs while glycine preferentially acts on NR2B-containing NMDARs^23^, this set of experiments further stresses that L-Lactate modulates NR2B-containing NMDARs.

Concerning the specificity of L-Lactate, the use of two of its analogues, Pyruvate and D-Lactate at 10 mM did not increase the Ca^2+^ signal evoked by the cocktail of glutamate/glycine (Fig. 2B). In fact, both molecules significantly decreased the Ca^2+^ responses by 52% (p < 0.05) and 51% (p < 0.05) respectively (Fig. 2B,C and Table 1), further stressing the specificity of L-Lactate in the potentiating the Ca^2+^ signal induced by the co-application of glutamate/glycine. In addition, when 10 mM L-Lactate was substituted with an equicaloric concentration of Glucose (5 mM extra Glucose ACSF), the Ca^2+^ signal evoked by the cocktail of glutamate/glycine is significantly decreased (control: 0.29 ± 0.04 a.u.; 5 mM extra Glucose: 0.14 ± 0.06 a.u.; n_cult_ = 8; n_cells_ = 425; p < 0.05), in a manner similar to that observed in the presence of Pyruvate (Fig. 2B,C and Table 1). This observation stresses again the specificity of L-Lactate for potentiation of NMDAR activity over other energy substrates.

### The L-Lactate-induced potentiation is dependent on the redox state of the cell

It has been shown that NMDARs express regulatory redox sites, the reduced state of them favoring NMDAR activity^16,17^. The metabolism of L-Lactate to Pyruvate by L-Lactate Dehydrogenase (LDH) results in the intracellular production of NADH, an endogenous reducing molecule. Consistently, in our cultures, exposure of neurons to ACSF containing L-Lactate (10 mM) significantly increases the intracellular NADH/NAD^+^ ratio by 68% (Control: 6.49 +/− 0.65; n_cult_ = 12; Lactate: 10.95 +/− 1.56; n_cult_ = 12; p < 0.005) (Table 2). This result is in agreement with the study of Yang *et al*.^6^ where they demonstrate that L-Lactate (but not Pyruvate) increases this intracellular NADH/NAD^+^ ratio in cultured neurons. We also tested Glucose as a possible source for the increase of the intracellular NADH/NAD^+^ ratio^24^. In the presence of 5 mM extra Glucose ACSF, the intracellular NADH/NAD^+^ ratio is significantly decreased by 29% (5 mM extra Glucose: 4.63 +/− 0.64; n_cult_ = 11; p < 0.05) (Table 2) confirming the specificity of L-Lactate to be the main source of production for cytosolic NADH, the endogenous reducing agent.

**Table 2:** Summary of intracellular NADH/NAD^+^ ratio measured in different conditions.

Condition	NADH/NAD^+^ ratio	One-Way Anova
Control (n_cult_ = 12)	6.49 +/− 0.65	
L-Lactate (10 mM) (n_cult_ = 12)	10.95 +/− 1.56	<0.005
5 mM extra Glucose (n_cult_ = 11)	4.63 +/− 0.64	<0.05

Values are means ± SEM. Statistics data correspond to a Dunnett’s post hoc test following a one-way ANOVA.

**Figure 3:**
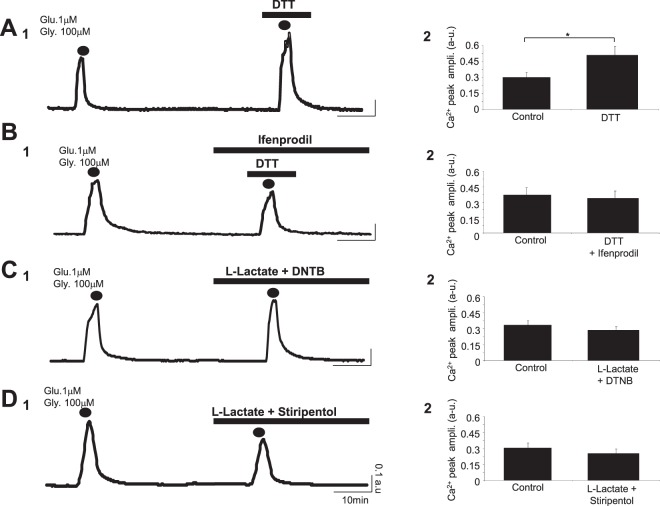
L-Lactate-induced potentiation depends on the redox state of the cell. (**A**_**1**_,**B**_**1**_,**C**_**1**_ and **D**_**1**_) Intracellular Ca^2+^ signals (average Fura-2 ratio) following 2 successive co-applications of glutamate/glycine (2 min; dots) and recorded from 4 different cultures in presence of DTT (1 mM) (n_cell_ = 43; **A**_**1**_), DTT (1 mM) + Ifenprodil (2 μM) (n_cell_ = 21; **B**_**1**_), L-Lactate (10 mM) + DTNB (200 μM) (n_cell_ = 62; **C**_**1**_) or L-Lactate (10 mM) + Stiripentol (200 μM) (n_cell_ = 42; **D**_**1**_). Only the reducing agent DTT alone potentiates the Ca^2+^ response evoked by the co-application of glutamate/glycine (**A**_**1**_). (**A**_**2**_,**B**_**2**_,**C**_**2**_ and **D**_**2**_) Bar charts summarizing the significant potentiating effect of DTT (n_cult_ = 9; n_cells_ = 343; **A**_**2**_) blocked by Ifenprodil (n_cult_ = 8; n_cells_ = 301; **B**_**2**_) and the blockade of the L-Lactate-induced potentiation in presence of DTNB (n_cult_ = 8; n_cells_ = 407; **C**_**2**_) or Stiripentol (n_cult_ = 9; n_cells_ = 298; **D**_**2**_) on the Ca^2+^ signal triggered by co-application of glutamate/glycine. Results are presented as means ± SEM (*p < 0.05; paired t-test).

Previous evidence has been provided showing that L-Lactate promotes plasticity gene expression by potentiating NMDAR signaling in neurons through a similar mechanism associated with changes in intracellular redox state^6^. Based on the foregoing we used DTT, a reducing agent, to test the possibility that the L-Lactate-induced potentiation of NMDAR activity is also associated with some changes in the redox state of the cell. In the presence of DTT (1 mM) the Ca^2+^ signal induced by the co-application of glutamate/glycine (1 μM/100 μM; 2 min) is strongly increased from 0.31 ± 0.04 a.u. in control to 0.52 ± 0.08 a.u. in DTT condition (p < 0.05; n_cult_ = 9; n_cells_ = 343) (Fig. 3A and Table 1). Moreover, in the presence of Ifenprodil (2 μM), DTT does not potentiate the Ca^2+^ response triggered by the glutamate/glycine cocktail (control: 0.37 ± 0.06 a.u.; DTT+ Ifenprodil: 0.33 ± 0.05 a.u.; n_cult_ = 8; n_cells_ = 301) (Fig. 3B and Table 1), this result reinforcing the notion of a similarity between the mechanism of action of L-Lactate and this reducing agent. Consistently, when L-Lactate is perfused in the presence of DTNB (200 μM), an oxidizing agent, the potentiation induced by L-Lactate on the Ca^2+^ response (control: 0.34 ± 0.06 a.u.; L-Lact. + DTNB: 0.32 ± 0.06 a.u.; n_cult_ = 8; n_cells_ = 407) is no longer observed (Fig. 3C and Table 1).

Finally, Stiripentol (200 μM), a blocker of Lactate Dehydrogenase (LDH) which prevents the metabolism of L-Lactate to Pyruvate and consequently the formation of NADH, also blocks the L-Lactate-induced potentiation (control: 0.31 ± 0.06 a.u.; L-Lact. + Stiripentol: 0.26 ± 0.05 a.u.; n_cult_ = 9; n_cells_ = 298) (Fig. 3D and Table 1), further stressing the fact that redox state changes evoked by L-Lactate through NADH production by LDH are essential for the potentiation of NMDAR activity.

### Blockade of the intracellular Ryanodine Receptor (RyR) impacts the maintenance of Ca^2+^ signal during L-Lactate-induced potentiation

**Figure 4:**
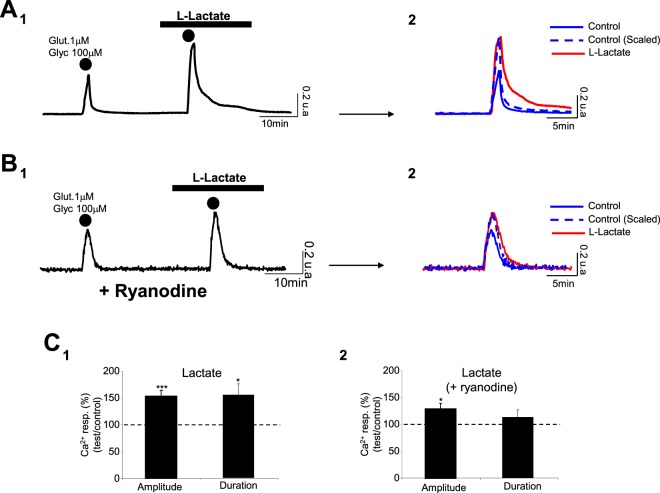
The RyRs blockade impacts only the maintenance of Ca^2+^ signal during L-Lactate-induced potentiation. (**A**_**1**_,**B**_**1**_) Averaged Ca^2+^ traces recorded from 65 neurons (**A**_**1**_) and 49 neurons (**B**_**1**_) following 2 successive applications of glutamate/glycine (2 min; dots) in control and in presence of L-Lactate (10 mM), the neurons in **B**_**1**_ coming from a culture pre-treated with ryanodine (100 μM). (**A**_**2**_,**B**_**2**_) The scaling of evoked Ca^2+^ responses obtained in (**A**_**1**_ and **B**_**1**_) shows that the L-Lactate-induced potentiation also corresponds to an increase of the decay time (in comparison with the scaled Ca^2+^ response obtained in control, **A**_**2**_), with ryanodine blocking this effect on the Ca^2+^ response kinetics (**B**_**2**_). (**C**_**1**_,**C**_**2**_) Bar charts summarizing the effect of L-Lactate on the Ca^2+^ signal triggered by co-application of glutamate/glycine on untreated cultures (n_cult_ = 11; n_cells_ = 468; **C**_**1**_) or cultures pre-treated with ryanodine (n_cult_ = 7; n_cells_ = 331; **C**_**2**_). Note that, when cultures are pre-treated with ryanodine, the potentiating effect of L-Lactate significantly persists only for the amplitude of the Ca^2+^ signal. Results are presented as means ± SEM (*p < 0.05; ***p < 0.005; paired t-test).

Obtained Results clearly indicate that the L-Lactate-induced potentiation is characterized not only by an increase in the Ca^2+^ peak amplitude but also by a longer peak duration (control: 551 ± 53 s; L-Lact.: 850 ± 114 s; n_cult_ = 11; n_cells_ = 468; p < 0.05) (Fig. 4A,C). We sought to determine whether the opening of NR2B-containing NMDARs alone could explain such an increase in Ca^2+^ response duration in L-Lactate condition or whether other activation cascades intervene in the generation of this Ca^2+^ signal. Thus, we investigated a possible involvement of Ca^2+^-induced Ca^2+^ release mediated by intracellular RyRs. Indeed, such an intracellular release of Ca^2+^ is: (1) a mechanism that can contribute to the amplification of the Ca^2+^ influx generated by NMDAR activation^25,26^ and (2) is known to be redox sensitive^27,28^. To this end, neuronal cultures were pre-treated with ryanodine at 100 μM to irreversibly block the intracellular RyR^29^. In these conditions, the L-Lactate-induced potentiation persists with a significant increase of Ca^2+^ peak amplitude of 30% (control: 0.32 ± 0.01 a.u.; L-Lact.: 0.41 ± 0.03 a.u.; p < 0.05; n_cult_ = 7; n_cells_ = 331) (Fig. 4B,C and Table 1). However, when cultures were pre-treated with ryanodine, the duration of the Ca^2+^ response is not significantly modified by L-Lactate (control: 487 ± 62 s; L-Lact.: 547 ± 69 s; n_cult_ = 7; n_cells_ = 331; p > 0.05) (Fig. 4B,C). This set of experiments suggests that the L-Lactate-induced potentiation is mainly due to a change of redox state of NR2B-containing NMDARs, resulting in Ca^2+^ influx which, in turn, can activate RyRs to amplify the Ca^2+^ signal.

### The increase of glutamate concentration triggers an NR2B component in the Ca^2+^ signal while L-Lactate-induced potentiation disappears

**Figure 5:**
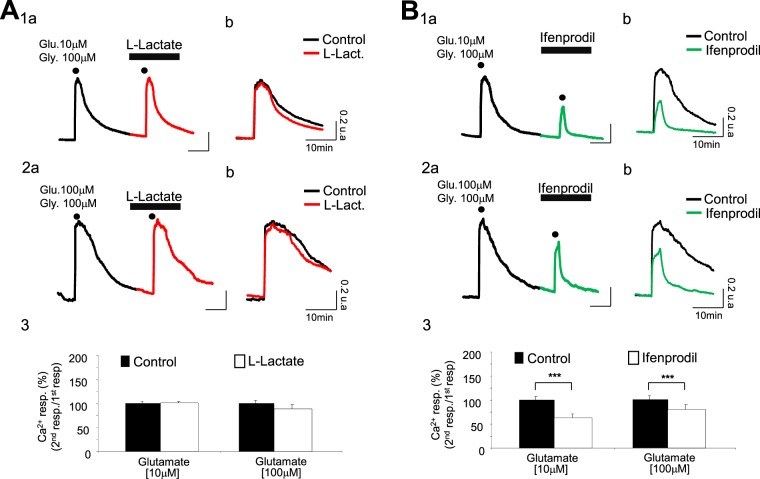
The L-Lactate-induced potentiation is blunted at increased glutamate concentration; activation of a NR2B component in the calcium response appears. (**A**_**1a**_ and **A**_**2a**_) Intracellular Ca^2+^ signals (black– and red-average Fura-2 ratio traces respectively for control condition and in presence of L-Lactate at 10 mM) recorded from 2 different cultures (**A**_**1a**_: n_cell_ = 54; **A**_**2a**_: n_cell_ = 29) and following 2 successive co-applications of glutamate/glycine (**A**_**1a**_: 10 μM/100 μM; **A**_**2a**_: 100 μM/100 μM; 2 min; dots). (**A**_**1b**_ and **A**_**2b**_) Expanded calcium traces shown in **A**_**1a**_ and **A**_**2a**_. In contrast to the Ca^2+^ response evoked by a weak glutamate concentration (Fig. 2), L-Lactate is inefficient at potentiating the Ca^2+^ response evoked by a strong concentration of glutamate. (**A**_**3**_) Bar charts summarizing the absence of potentiation by L-Lactate of Ca^2+^ responses evoked by the co-application of glutamate/glycine when concentration of glutamate is 10 μM (n_cult_ = 6; n_cells_ = 276) or 100 μM (n_cult_ = 6; n_cells_ = 155). (**B**_**1a**_ and **B**_**2a**_) Intracellular Ca^2+^ signals (black– and green-average Fura-2 ratio traces respectively for control condition and in presence of Ifenprodil at 2 μM) recorded from 2 different cultures (**B**_**1a**_: n_cell_ = 31; **B**_**2a**_: n_cell_ = 27) and following 2 successive co-applications of glutamate/glycine (**B**_**1a**_: 10 μM/100 μM; **B**_**2a**_: 100 μM/100 μM; 2 min; dots). (**B**_**1b**_ and **B**_**2b**_) Expanded calcium traces shown in **B**_**1a**_ and **B**_**1b**_. They clearly indicate a strong decrease of the Ca^2+^ response evoked by the co-application of glutamate/glycine in presence of Ifenprodil. (**B**_**3**_) Bar charts summarizing the significant blocking effect of Ifenprodil on Ca^2+^ responses evoked by the co-application of glutamate/glycine when concentration of glutamate is 10 μM (n_cult_ = 6; n_cells_ = 187) or 100 μM (n_cult_ = 6; n_cells_ = 138). Results are presented as means ± SEM (***p < 0.001; paired t-test).

The experiments reported so far were carried out in the presence of a low concentration of glutamate (1 μM), and of glycine (100 μM). Considering that the concentration of glutamate can reach values anywhere from 30 μM up to over 1 mM in the synaptic cleft during neuronal activation^21^, we decided to determine if the L-Lactate-induced potentiation persisted at higher concentrations of glutamate. When applying 2 successive co-applications of glutamate/glycine (10 μM/100 μM; 2 min) according to the same protocol, we observed no difference between the two evoked Ca^2+^ signals in control and L-Lactate conditions with respectively 0.49 ± 0.02 a.u. and 0.51 ± 0.03 a.u. for the Ca^2+^ peak amplitude (n_cult_ = 6; n_cells_ = 276) (Fig. 5A and Table 1). Similarly, with a glutamate/glycine cocktail of 100 μM/100 μM (2 min), the two evoked Ca^2+^ signals in control and L-Lactate conditions are of the same order (control: 0.55 ± 0.05 a.u.; L-Lact: 0.49 ± 0.07 a.u.; n_cult_ = 6; n_cells_ = 155) (Fig. 5A and Table 1), indicating that L-Lactate is unable to potentiate the NMDAR activity at high concentrations of glutamate.

Interestingly, we have previously observed that a low concentration of glutamate (1 μM), even in association to glycine, was unable to activate NR2B-containing NMDARs, except in the presence of L-Lactate. The fact that the L-Lactate-induced potentiation disappears in the presence of higher glutamate concentrations (always in presence of glycine), suggests that NR2B-containing NMDARs are already stimulated by higher glutamate concentrations, making the action of L-Lactate ineffective. To address this question, we used Ifenprodil in the presence of the above mentioned higher concentrations of glutamate. As shown in Fig. 5B, the Ca^2+^ signal evoked by 10 μM glutamate in the presence of 100 μM glycine (2 min) is strongly and significantly reduced by Ifenprodil (2 μM) (control: 0.52 ± 0.06 a.u.; Ifenprodil: 0.32 ± 0.06 a.u.; p < 0.005; n_cult_ = 6; n_cells_ = 187) (Table 1). The same type of antagonism by Ifenprodil is observed for a co-application of glutamate/glycine (100 μM/100 μM): the amplitude of the Ca^2+^ response triggered by glutamate/glycine is 0.59 ± 0.08 a.u. in control condition and 0.42 ± 0.09 a.u. with Ifenprodil (n_cult_ = 6; n_cells_ = 138) (Fig. 5B and Table 1).

This set of results indicates that in the presence of high concentrations of glutamate (+glycine), NR2B-containing NMDARs are stimulated, and that L-Lactate renders low concentrations of glutamate effective through these same NR2B-containing NMDARs.

### L-Lactate decreases the rate of cell death triggered by pathological concentrations of glutamate: involvement of the Pyruvate/ATP pathway

**Figure 6:**
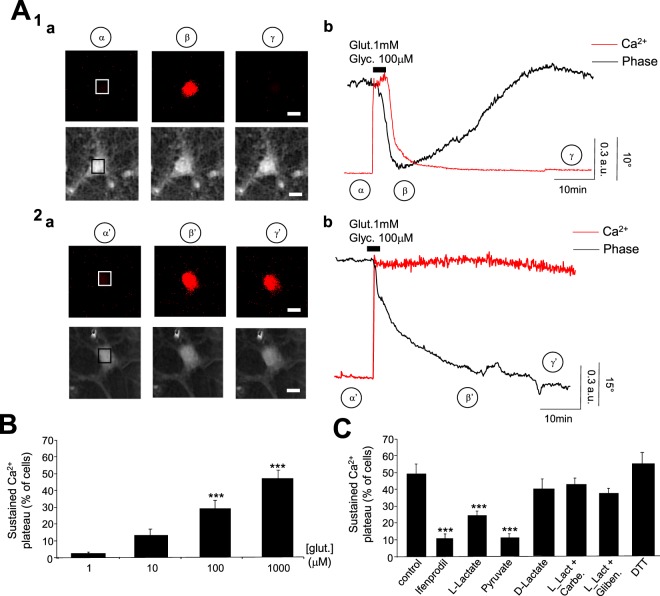
L-Lactate decreases the rate of cell death triggered by excito-toxic concentration of glutamate. (**A**) Two representative reversible (**A**_**1**_) and non-reversible (**A**_**2**_) Ca^2+^ and QPS recorded from two different neurons after an excito-toxic co-application of glutamate/glycine (1 mM/100 μM; 2 min). (**A**_**1a**_ and **A**_**2a**_) Fluorescence (top row) and QPS (below) images of neurons before (α & α′), 15 min after (β & β′) and 1 h after (γ & γ′) co-application of glutamate/glycine (Scale bar: 20 μm). The squares in the middle of cells correspond to the regions of interest where the fluorescent (Fura-2; Ratio) and QPS are measured. (**A**_**1b**_ and **A**_**2b**_) Traces recorded in Fluorescence (red line) and in DHM (black line) from neurons in (**A**_**1a**_ and **A**_**2a**_). For the neuron in **A**_**1**_, the fluorescent trace indicates a transient Ca^2+^ signal with a complete recovery concomitant to a reversible decrease of the QPS that are associated with recovery (non-cell death). In contrast, traces recorded in Fluorescence (red line) and in DHM (black line) from the neuron in **A**_**2a**_ show a sustained Ca^2+^-plateau associated with an irreversible decrease of the QPS associated with a cell death process. (**B**) Bar charts summarizing the percentage of cells displaying a sustained Ca^2+^ -plateau for different concentrations of glutamate (1 μM: n_cult_ = 9; n_cells_ = 275; 10 μM: n_cult_ = 6; n_cells_ = 276; 100 μM: n_cult_ = 6; n_cells_ = 155; 1 mM: n_cult_ = 7; n_cells_ = 263). This difference becomes significant for glutamate concentrations from 100 μM. (**C**) The bar chart shows the percentage of glutamate/glycine-induced Ca^2+^ sustained plateau obtained in different conditions: control (n_cult_ = 7; n_cells_ = 263), Ifenprodil (2 μM; n_cult_ = 6; n_cells_ = 260), L-Lactate (10 mM; n_cult_ = 8; n_cells_ = 282), Pyruvate (10 mM; n_cult_ = 6; n_cells_ = 264), D-Lactate (10 mM; n_cult_ = 6; n_cells_ = 246), L-Lactate (10 mM) + Carbenoxolone (10 μM) (n_cult_ = 5; n_cells_ = 164), L-Lactate (10 mM) + glibenclamide (200 μM) (n_cult_ = 4; n_cells_ = 141) and DTT (1 mM; n_cult_ = 6; n_cells_ = 154). Only L-Lactate, Pyruvate and Ifenprodil significantly decrease the percentage of cells displaying a sustained Ca^2+^-plateau. Results are presented as means ± SEM (***p < 0.005, One-way Anova/post-test “Dunnett’s” ).

It is well established that an excessive concentration of glutamate overstimulates NMDARs triggering several downstream neurotoxic cascades caused by an intracellular Ca^2+^ overload^30^. As expected, a co-application of glutamate/glycine (1 mM/100 μM; 2 min) triggers strong Ca^2+^ signals with an amplitude of 0.87 ± 0.14 a.u. (n_cult_ = 7; n_cells_ = 263) that entails two components (Fig. 6A and Table 3): 1/ a reversible response corresponding to a Ca^2+^ peak followed by a complete or incomplete recovery; 2/ an irreversible response corresponding to a sustained plateau of the Ca^2+^ signal. These two Ca^2+^ responses were observed in 135 out of 263 cells (around 51%) and 128 out of 263 cells (around 49%), respectively. Concomitantly to the Ca^2+^ overload, it has been shown that glutamate-induced excito-toxicity triggers a rapid and acute swelling of cell bodies due to Na^+^, Cl^−^ and water inflows^31,32^. Accordingly, by using a multi-modality imaging system combining Ca^2+^ imaging with Digital Holographic Microscopy (DHM), a technique that allows to detect early-stages of neuronal excito-toxic death^14,33,34^, we observed that 111 out of 135 cells (around 82%) displaying a transient Ca^2+^ response also exhibit a reversible decrease of the quantitative phase signal (QPS) (Δφ = −22.5 ± 5.7°), this optical signal being synonymous with cell survival. In contrast 106 out of 128 cells (around 83%) displaying a sustained plateau of Ca^2+^ signal exhibit an irreversible decrease of QPS (Δφ = −31.1 ± 7.4°), the latter being associated with the process of cell death^14,33^. This sustained plateau of the Ca^2+^ signal, which is a cell death marker, can be observed at a concentration of glutamate of 100 μM (p < 0.005) (Fig. 6B).

**Table 3:** Summary of the rate of sustained plateau of Ca^2+^ signal (cell death process) induced by glutamate in different conditions.

Glutamate/Glycine (1 mM/100 μM; 2 min)	% Ca^2+^ sustained plateau (Cell death process)	One-Way Anova
Control (n_cult_ = 7; n_cells_ = 263)	51.4 ± 6.7	
Ifenprodil (2 μM) (n_cult_ = 6; n_cells_ = 260)	9.7 ± 3.4	<0.005
L-Lactate (2 mM) (n_cult_ = 6; n_cells_ = 338)	42.6 ± 7.8	*n*.*s*
L-Lactate (5 mM) (n_cult_ = 6; n_cells_ = 302)	28.7 ± 4.9	<0.05
L-Lactate (10 mM) (n_cult_ = 8; n_cells_ = 282)	22.7 ± 3.7	<0.005
Pyruvate (10 mM) (n_cult_ = 6; n_cells_ = 264)	9.0 ± 3.7	<0.005
D-Lactate (10 mM) (n_cult_ = 6; n_cells_ = 246)	38.5 ± 7.4	*n*.*s*
L-Lactate (10 mM) + carbenoxolone (10 μM) (n_cult_ = 5; n_cells_ = 164)	42.8 ± 3.5	*n*.*s*
L-Lactate (10 mM) + glibenclamide (200 μM) (n_cult_ = 4; n_cells_ = 141)	36.0 ± 4.0	*n*.*s*
DTT (1 mM) (n_cult_ = 6; n_cells_ = 154)	54.0 ± 7.5	*n*.*s*

Values are means ± SEM. Statistics data correspond to a Dunnett’s post hoc test following a one-way ANOVA (*n*.*s*: non significant, p > 0.05).

The cell death process is largely attributable to the activation of NR2B-containing NMDARs as the amplitude (0.57 ± 0.07 a.u) and the rate of cells displaying a sustained plateau of Ca^2+^ signal (9.7 ± 3.4%) are significantly decreased (<0.005 for the two parameters) by Ifenprodil (2 μM) (n_cult_ = 6; n_cells_ = 260) (Fig. 6C and Table 3). In the presence of L-Lactate (10 mM), the amplitude of the Ca^2+^ signals triggered by the mostly excito-toxic co-application of glycine/glutamate (1 mM/100 μM; 2 min) is equivalent to the control condition (0.88 ± 0.22 a.u.; n_cult_ = 8; n_cells_ = 282) confirming that, for strong concentrations of glutamate, L-Lactate does not potentiate the Ca^2+^ signal. However, under the same conditions, the percentage of cells displaying a sustained plateau for the Ca^2+^ signal is significantly decreased in the presence of L-Lactate (22.7 ± 3.7%; p < 0.005) (Fig. 6C and Table 3). Interestingly, this L-Lactate-induced neuroprotection pathway is mimicked by Pyruvate (9 ± 3.7% of cells displaying sustained plateau of Ca^2+^ signal; n_cult_ = 6; n_cells_ = 264) but not by D-Lactate (38.5 ± 7.4% of cells displaying sustained plateau of Ca^2+^ signal; n_cult_ = 6; n_cells_ = 246) (Fig. 6C and Table 3). Moreover, when pannexins are inactivated with carbenoxolone at 10 μM or ATP-sensitive potassium channels (K_ATP_) with glibenclamide (200 μM), the L-Lactate-induced neuroprotection disappears with only 42.8 ± 3.5% (n_cult_ = 5; n_cells_ = 164) and 36 ± 4% (n_cult_ = 4; n_cells_ = 141) of cells displaying sustained plateau of Ca^2+^ signal respectively (Fig. 6C and Table 3). These results are in agreement with our recent study showing that the neuro-protective properties of L-Lactate against a glutamate excito-toxic insult are associated with a metabolic pathway linked to ATP production, release and activation of a P2Y2/K_ATP_ cascade^14^. In contrast, the NADH pathway that mediates the potentiating action of L-Lactate on low glutamate concentrations is not involved since the reducing agent DTT (1 mM) has no neuroprotective effect (54 ± 7.5% of cells displaying sustained plateau of Ca^2+^ signal; n_cult_ = 6; n_cells_ = 154) (Fig. 6C and Table 3).

## Discussion

**Figure 7:**
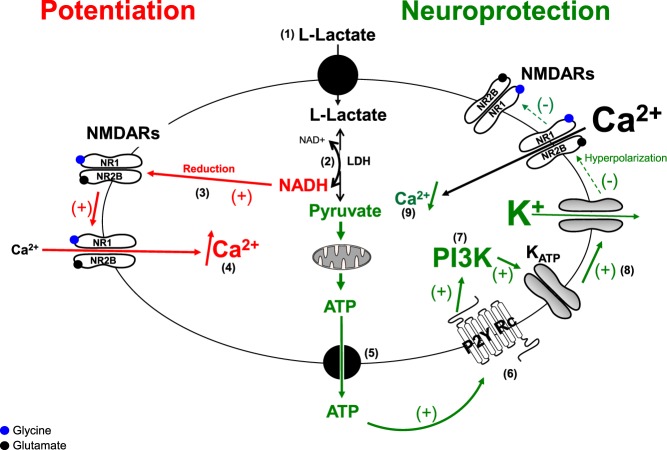
Schematic representation of the dual effect of L-Lactate. Extracellular L-Lactate is transported into neurons (1) and is metabolized by Lactate Dehydrogenase (LDH) in presence of NAD^+^ (2) to produce Pyruvate and NADH. Pyruvate and NADH are engaged in two distinct and independent pathways: Potentiation Pathway (or NADH Pathway in red): For a subthreshold concentration of glutamate unable to activate NR2B-containing NMDARs, NADH acts as reducing agent (3) to allow the opening of the NR2B-containing NMDARs’ ion channel resulting in a Ca^2+^ influx (4). Neuroprotective Pathway (or Pyruvate/ATP Pathway in green): When glutamate over-activity triggers a strong inflow of Ca^2+^ through the NR2B-containing NMDARs, Pyruvate is metabolized into the mitochondria to produce ATP then released through pannexins (5) to act on the metabotropic purinergic receptor P2Y2 in an autocrine/paracine manner (6). Stimulation of P2Y2 receptors activates the PI3K pathway (7) which, in turn, elicits the opening of K_ATP_ channels, hence leading to hyperpolarization of neurons (8), the consequence being a decrease in neuronal excitability leading to neuroprotection (decrease of the Ca^2+^ influx through the closure of NMDARs) (9). Points 6 and 7 have been presented in Jourdain *et al*.^14^.

In recent years two apparently contrasting effects of L-Lactate have been demonstrated: the potentiation of the NMDAR activity resulting in plasticity gene expression^6^ and the neuroprotective effect against excessive NMDAR activity^14^, the question being then how the same molecule can potentiate some positive effects of glutamate while inhibiting others, both involving NMDARs? In this report, we show that this apparent paradox is explained by the efficiency of L-Lactate to potentiate NMDARs for a certain activation window corresponding to a subthreshold concentration of glutamate. Indeed, the data reported here clearly show that L-Lactate potentiates the NMDAR activity only for 1 μM of glutamate, a concentration in the range of its EC50 for NMDARs (around 0.5–5 μM). In contrast, the L-Lactate-induced potentiation disappears for concentrations ranging between 10 μM to 1 mM of glutamate, thus excluding any direct role of L-Lactate in the excessive activation of NMDARs during an excito-toxic episode. This “activity switch” of L-Lactate results in its neuroprotective properties to significantly decrease the cell death process against an exposure to high concentrations of glutamate (and glycine) through a complex metabolic pathway involving the synthesis of Pyruvate, ATP release (through the opening of pannexins) and subsequent activation of the ATP-dependent potassium channel K_ATP_ as shown previously^14^. The only common point between the potentiation and neuroprotective pathways is L-Lactate itself while the two products resulting from its metabolism namely NADH and ATP, are involved in potentiation (NADH) and in neuroprotection (ATP) (Fig. 7). Most importantly, there is no interconnection between these two pathways as revealed by the fact that:

1. Pyruvate is neuroprotective but unable to potentiate NMDAR activity, in fact decreasing it, probably because it decreases the neuronal excitability through the final activation of K_ATP_ channels^14^. Indeed, as NMDARs are open under a depolarized membrane potential, activation of K_ATP_ channels will result in a decrease of excitability counteracting NMDAR activation. This mechanism also explains why Pyruvate decreases the Ca^2+^ signal evoked by a low concentration of glutamate. A notable point concerns D-Lactate which also decreases the Ca^2+^ signal potentiated by L-Lactate in the presence of low concentrations of glutamate. Indeed, D-Lactate is a non-metabolizable analogue of L-Lactate without apparent action on the K_ATP_, unlike Pyruvate^14^. However, D-Lactate has been shown to strongly reduce neuronal activity by acting on the G-protein-coupled Lactate receptors, GPR81 (HCA1)^35^ which in turn decreases the excitability of the neuronal network and, by consequence, the probability to activate NMDARs by a subliminal concentration of glutamate. However, in contrast to Pyruvate, D-Lactate is not efficient to protect neurons against excito-toxic insults *in vitro*^14^.
2. The reducing agent DTT which presumably mimics the effect of NADH, by affecting redox-sensitive processes, potentiates NMDAR activity without neuroprotective properties, a result in agreement with literature since the reduced state of NMDARs stimulates its own activity^16,17^, in particular evoked by DTT^18,36^. Here it should be noted that the potentiation of Ca^2+^ signal induced by L-Lactate is slightly lower than the DTT-induced potentiation (+52% *vs* +69%), a result consistent with the fact that the pure potentiation activity of L-Lactate is partly counteracted by its involvement in the Pyruvate pathway (decrease of membrane excitability).

Our results clearly show that extracellular application of L-Lactate produces an increase of the intracellular NADH/NAD^+^ ratio confirming the relationship between the cytosolic NADH production originating from L-Lactate metabolism and L-Lactate-induced NMDAR potentiation of the Ca^2+^ signal. In contrast, the 5 mM extra Glucose ACSF has no effect on the intracellular NADH/NAD^+^ ratio and, in parallel, decreases the Ca^2+^ signal induced by the glutamate/glycine cocktail. Such a result is consistent with the work of Porras *et al*.^37^ showing that glutamate does not trigger Glucose utilization but rather inhibits Glucose transport and utilization in neurons. In contrast, other studies showed that neuronal depolarization is accompanied by an increase in cytosolic NADH/NAD^+^ ratio that is associated with a reduction of the malate-aspartate shuttle activity through the glycolytic pathway^38^. It was also observed that Glucose utilization is positively correlated with intracellular Ca^2+^ signaling whereas L-Lactate utilization is not^24,39^. Our observation that the inhibition of LDH by stiripentol is sufficient to block the L-Lactate-induced potentiation does not argue in favor of a role of the modulation of malate-aspartate shuttle as source of NADH in the potentiation of Ca^2+^ signal associated to NMDAR activity. Moreover, this Glucose-induced Ca^2+^ signaling seems to be effective for 100–300 μM of NMDA^39,40^. This range of NMDA concentrations being 4 to 10 fold higher than the EC50 for NMDARs expressing the NR2B sub-unit^41^, it is conceivable that, at 100–300 μM of NMDA, all of NR2B-containing NMDARs are activated by these high concentrations of NMDA, a situation that does not require L-Lactate which is only active on “silent” NR2B-containing NMDARs. Besides, our results clearly show that, at high concentrations of glutamate (or NMDA), L-Lactate would tend to decrease the amplitude of the Ca^2+^ signal which may explain why Glucose (and not L-Lactate) is positively correlated with intracellular Ca^2+^ signaling when NMDA is around 100–300 μM^24,39^. In conclusion, while we cannot exclude glycolysis (*i*.*e*. Glucose metabolism) and the malate-aspartate shuttle as possible metabolic pathways related to intracellular NADH production in neurons, our results demonstrate that L-Lactate metabolism (through LDH activity) is the main pathway involved in the production of cytosolic NADH allowing the potentiation of Ca^2+^ signal associated with NMDAR activity.

Concerning more specifically the redox state of the cell, the presence of redox sites located on the extracellular part of NMDARs on the NR1 subunit has been demonstrated^17^. We have observed that bath application of DTT, a reducing agent, induces a potentiation of NMDAR activity suggesting a possible extracellular action of this agent. Furthermore, bath application of DTNB, an oxidizing agent considered as membrane impermeant^42^, blocks the L-Lactate-induced potentiation strengthening the view that extracellular redox-sensitive sites on NMDARs are being affected. Of note is the fact that the L-Lactate-induced potentiation is also blocked by stiripentol, an inhibitor of the intracellular enzyme LDH involved in the conversion of Lactate to Pyruvate and, by consequence, in the regulation of the intracellular NADH/NAD^+^ ratio. This consideration would indicate that the changes in redox state evoked by the changes in the NADH/NAD^+^ ratio are likely to operate intracellularly as a primary site of action. However, extracellular sites of action of intracellular-produced NADH acting on extracellular redox-sensitive NMDAR sites could be considered. Indeed, it has been suggested that NADH (and NAD^+^) can be transported across the plasma membrane^43^ via the purinergic P2X7 receptor^44^, a receptor also expressed in neurons^45,46^. Thus it is conceivable that intracellularly formed NADH is released through P2X7 receptors and acts on extracellular redox-sensitive sites of the NMDAR. Further work will be needed to confirm or invalidate this hypothesis. Another additional mechanism of red/ox-dependent NMDAR activity modulation by L-Lactate is related to the intracellular RyRs known to be involved in the release of Ca^2+^ from intracellular stores, which expresses a few cysteine residues highly susceptible to oxidative modifications^28^. Nevertheless, the fact that L-Lactate-induced NMDAR-dependent potentiation of the Ca^2+^ peak amplitude persists despite the blockade of RyRs provides evidence that RyRs are not the main target of the redox change initiated by L-Lactate. Nevertheless, our results confirm that RyRs contribute to the maintenance of the Ca^2+^ influx generated by NMDAR activation^25,26^.

An additional question concerns the nature of NR2 subunit expressed in NMDARs which is potentiated by L-Lactate. Our data show that, in control conditions, the Ca^2+^ signal evoked by a low concentration of glutamate (1 μM), in association with a saturating concentration of glycine (100 μM), is not due to activation of NR2B-containing NMDARs since Ifenprodil, the specific blocker of the NR2B sub-unit, is ineffective in blocking this Ca^2+^ signal. Since NR2A and NR2B subunits are more strongly expressed than NR2C and NR2D subunits in cortical neuronal cultures^47^ it is likely that the Ca^2+^ signal evoked in control conditions is mainly due to an activation of NR2A-containing NMDARs. In the presence of L-Lactate, an additional Ca^2+^ component appears. Indeed, as shown in Fig. 2A, this additional Ca^2+^ component is associated with an activation of NR2B-containing NMDARs since not only it is blocked by Ifenprodil but also L-Lactate is less efficient at potentiating the evoked Ca^2+^ signal when glutamate is co-applied with D-Serine, the analogue of glycine purported to be more specific to the NR2A sub-unit^23^. Moreover, we still observe this L-Lactate-induced potentiation when we substitute 1 μM glutamate with 30 μM of NMDA, this NMDA concentration corresponding to its EC50 for NMDARs expressing the NR2B sub-unit^41^. Finally, the additional Ca^2+^ component induced by DTT is also sensitive to Ifenprodil, a result in agreement with the fact that NR2B subunit has an additional redox site involving a more long-lasting effect of reducing agents for NR1/NR2B than for NR1/NR2A^48^.

We cannot exclude a possible potentiating action of L-Lactate on NR2A-containing NMDARs. However the location of these NR2A-containing NMDARs, essentially in postsynaptic membrane^49^, implies that they are exposed to high (saturating) concentrations of glutamate in the synaptic cleft during an excitatory stimulus, up to 1 mM during an action potential^50^. In contrast, the NMDARs expressing the NR2B sub-unit are thought to be localized pre- or extra-synaptically^51,52^. Reported values of baseline glutamate concentrations in extracellular space at these sites range between 0.02 to 5 μm *in vitro* or *in vivo*^21,53^. Furthermore these concentrations remain relatively stable because of the rapid uptake of glutamate by astrocytic glutamate transporters even during high frequency stimulation^54,55^. Finally, reports have shown that NR2B-containing NMDARs are associated with adjacent astrocytic processes^56^, allowing for modulation of synaptic activity^56,57^ and enabling the synchronization of neural networks through the genesis of large Slow Inward Currents^58,59^.

It has also been shown recently that NR2B-containing NMDARs are necessary for the formation of Long Term Potentiation^60^. This observation, taken with the demonstration that L-Lactate transport from astrocytes to neurons is necessary for LTP and memory consolidation^9^ raises some heuristically interesting considerations. The observation that L-Lactate potentiates NMDAR activity as shown in the present article concerning Ca^2+^ signaling and in Yang *et al*.^6^ concerning the induction of plasticity genes should be placed in the context of *in vivo* studies showing that L-lactate produced from astrocytic glycogen is necessary for memory consolidation^9,10^. Indeed glycogenolysis in astrocytes resulting in L-Lactate formation^61^ is dependent on beta2-adrenergic receptor activation^10^. The release of L-Lactate and its uptake into neurons via MCT2 transporters promotes plasticity genes expression and is necessary for memory consolidation. One of the behavioral modalities during which the Locus Coeruleus, the main source of noradrenergic fibers projecting to the hippocampus and neocortex, is the attentional regime that accompanies learning^62^. Thus, the behavioral modality that would result in the potentiation of NMDAR signaling by L-lactate would indeed be the attention- and learning-mediated release of the glycogenolytic neuromodulator noradrenaline onto glycogen-containing astrocytes in the cortex. It is thus reasonable to hypothesize that an astrocytic release of L-Lactate after a noradrenergic stimulation can activate some pre- or extra-synaptic NR2B-containing NMDARs which are normally “silent” because the ambient concentration of glutamate is very low (<1 μM). In turn, the activation of such pre- or extra-synaptic NR2B-containing NMDARs can modulate the threshold of the induction of Long Term Potentiation. Such a sequence of events is consistent with the modulatory role of L-Lactate on neuronal plasticity and Long-Term Memory formation^9^. Because of its slower kinetics causing stronger Ca^2+^ influx^51^, the NR2B sub-unit is also involved in extra-synaptic NMDAR-dependent death signaling^63^ consistent with our results showing that Ifenprodil strongly and significantly decreases the rate of cell death induced by an excito-toxic application of glutamate.

Finally, this dual action of L-Lactate on the activity of NR2B-containing NMDARs is also dependent on the extracellular L-Lactate concentration. The fact that the efficient concentrations of L-Lactate are different for potentiation and/or neuroprotection (Tables 1 and 3) reinforces the notion of two independent pathways for L-Lactate. As it is the case for glutamate whose concentration may range between 1 μM and 1 mM, notably in the synaptic cleft during neuronal activation^21^, it is likely that L-Lactate can also reach high concentrations locally. Indeed, considering a width of the extracellular space between neurons and astrocytes ranging from 50 nm to 3.2 µm^64^ and the fact, that the astrocytic release of L-Lactate is faster (>10^5^ Lactate molecules per second) than the slow L-Lactate re-uptake by the neuronal MCTs (around 10^2^ Lactate molecules per second)^65^ it is likely that the extracellular L-Lactate concentrations can locally reach concentrations of 10 mM and even higher in the vicinity of the synapse.

In conclusion, as summarized in Fig. 7, the present results demonstrate the dual and at first analysis paradoxical properties of L-Lactate to potentiate and to protect respectively neuronal networks. The occurrence of each effect is dependent on the strength of the glutamatergic stimulus to activate (or not) pre- or extra-synaptic NR2B-containing NMDARs. To our knowledge, there are no or few molecules with these dual properties which make L-Lactate a potentially attractive candidate for clinical and pharmacological research in particular for nervous system pathologies such as stroke, spinal cord injury and progressive neurodegenerative diseases.

## Materials and Methods

Experiments were conducted in accordance with the Swiss Federal Guidelines for Animal Experimentation and were approved by the Cantonal Veterinary Office for Animal Experimentation (Vaud, Switzerland).

### Cell culture preparation

Primary cultures of cortical neurons were prepared from embryonic day 17 (E17) OF1 mice embryos (Charles River Laboratories, L’Arbresle, France) as previously described^6^. Neurons were plated on 25 mM poly-L-Ornithine (Sigma) coated coverslips at an average density of 4 × 10^4^ cells/cm^2^ and maintained in Neurobasal medium (Gibco, containing 25 mM Glucose) supplemented with B27 (Gibco), GlutaMAX, penicillin (50 U/mL) and streptomycin (50 µg/mL) (Invitrogen, Basel, Switzerland) at 37 °C in a humidified atmosphere containing 5% CO_2_ and 95% air and were used at day *in vitro* (DIV) 17 to 22. These culture conditions typically produced 93% pure neuronal cultures, as assessed by microtubule-associated protein 2 (MAP2, neuronal marker) and glial fibrillary acidic protein (GFAP, astrocytic marker) co-immunostaining^66^.

### Cell loading

Cells are loaded with Fura-2-AM (Fura-2, Invitrogen) in order to measure intracellular [Ca^2+^]. Fura-2 is diluted in DMSO at stock concentrations of 2 mM. 2 μl of this stock are then added to 1 ml of perfusion medium with 1% Bovine Serum Albumine (BSA, Sigma) added to improve uptake of the dye. Cells were loaded in this solution for 30 minutes at 37 °C. For experiments with Ryanodine (Tocris), the blocker is added to the loading solution at 100 μM during 30 minutes. In these conditions, the effect of Ryanodine is irreversible^29^.

### Optical set-up

Microscopy was performed on a DHM T1000 microscope (Lyncée Tec SA) with an added custom fluorescence module^67^. The DHM component of the measurement system consists in a standard transmission DHM setup^68,69^ enabling the monitoring of cells in time through phase measurement as described in other studies^33,67^. In short, the light emitted by a laser diode (λ = 684 nm), is split into two beams: The object beam interacts with the specimen, and the scattered light is collected by a 10x microscope objective having a numerical aperture of 0.3 in air (Leica Microsystems, Fluotar). This beam forms an interference pattern with the reference beam which is recorded by a CCD camera (8-bit, pixel size 6.45 μm) in the Fresnel regime. Briefly, quantitative phase signals (QPS) using QP-DHM is an interferometric imaging technique that allows to visualize^69^ transparent specimens, including living cells, by measuring the phase retardation induced by the specimens on a transmitted wave (for a more detailed description of the basic design of the imaging system, see Marquet *et al*.^69^). In the context of living cells recordings by QP-DHM, the phase retardation or QPS, expressed as Δφ, is highly sensitive to the intracellular refractive index, which mainly depends on protein content^70^. Consequently, any transmembrane water movement, modifying the intracellular refractive index through a dilution or concentration process, drastically alters the QPS^70^. Therefore, a decrease of QPS corresponds to an inflow of water while an increase of phase shift indicates an exit of water^33,70^.

The fluorescence excitation light is provided by a monochromator (Polychrome V, Till Photonics), delivering light ranging from 320 nm to 680 nm, with a power of typically 10 mW at 470 nm and a bandwidth of 15 nm. Employing a monochromator enables the possibility of rapidly switching the excitation wavelength without using any excitation filter, thus avoiding any mechanical movement. The ratiometric dye Fura-2 is excited at both λ_1_ = 340 nm and λ_2_ = 380 nm. In order to enable excitation for Fura-2, a dichroic mirror with a cutting wavelength of 409 nm is used to send the excitation light to the specimen, while the fluorescence emission is filtered at 510 nm with 84 nm bandwidth. The fluorescence light is then detected by a CCD camera (INFINITY3S-1, Lumenera Corporation) recording 16-bit images with an exposure time of 800 ms.

The extraction of the two different signals of fluorescence and DHM was performed by employing two different dichroic mirrors, enabling first the separation of the line wavelength employed for digital holography, and second to enable the epifluorescence excitation^67^.

### Fluorescence measurements

Fluorescence images are acquired with the Micro-Manager software (as described in Edelstein *et al*.^71^), at a frame rate of 0.15 Hz. Temporal signals are then extracted from images by computing the mean value of the fluorescence intensity within the cell soma. A baseline, measured as the average signal of 5 empty regions in the field of view, is then subtracted from all signals to suppress the background contribution. Signals from Fura-2 are then computed as ratios *R* of the two excitation wavelengths (F_340_/F_380_).

### Phase signal measurements

Holograms are acquired at a frame rate of 0.1 Hz during all experiments. Quantitative phase images are then retrieved from measured holograms by employing standard reconstruction methods for off-axis holograms^68,72^ as provided by the Koala software (Lyncée Tec SA). Temporal phase signals are then extracted from images by monitoring the mean phase value within the cell soma, where a background signal is also subtracted from an empty region in the field of view to suppress potential drifts in the signal. An extensive quality control of the DHM technique has been published by Rappaz *et al*.^73^.

### Experimental protocol

Coverslips were mounted on a closed perfusion chamber used to apply different media to the cells. Standard media (ACSF) consisted of HEPES-buffered standard physiological perfusion medium^14^ and containing NaCl 140 mM, KCl 3 mM, CaCl_2_ 3 mM, MgCl_2_ 2 mM, Glucose 5 mM, HEPES 10 mM, adjusted to a pH of 7.3 with NaOH.

All experiments were performed at room temperature. Glutamate, Glycine, D-Serine, L-Lactate, D-Lactate, Pyruvate, MK801, Ifenprodil, dithiothreitol (DTT), 5,5′-dithiobis(2-nitrobenzoic acid) (DTNB), Stiripentol were provided by Sigma-Aldrich and added to ACSF. For most experiments, 2 successive pulses of glutamate (1–100 μM) and glycine (100 μM) are applied to the cells spaced out by a minimum delay of 20 min. The amplitude of the 1^st^ and the 2^nd^ Ca^2+^ response induced by the glutamate/glycine cocktail are compared. For the experiments performed at 1 mM of glutamate (+100 μM of glycine), only a single pulse is applied due to a higher number of excito-toxic responses. Finally, for some experiments, 5 mM of Glucose are added to the ACSF (final concentration of Glucose: 10 mM; annotated 5 mM extra Glucose ACSF).

### NADH/NAD^+^ assay

Cycling assays for nicotinamide adenine dinucleotides were adapted from previously published methods^74,75^. Cells were pre-incubated in ACSF during 30 min at 37 °C and subsequently switched to normal ACSF (control), L-Lactate-containing ACSF or 5 mM extra Glucose ACSF for an extra 10 min at 37 °C. Then cells were rinsed twice with ice-cold PBS, harvested in 400 μL ice-cold carbonate-bicarbonate buffer (100 mM Na_2_CO_3_, 20 mM NaHCO_3_ containing 10 mM Nicotinamide to inhibit NADase), and frozen at −80 °C. Cell membranes were lysed by heat-shock in a 37 °C water bath and immediately chilled on ice. Extracts were centrifuged at 12,000 × *g* for 30 min at 4 °C and half of the supernatant was heated at 60 °C for 30 min to denature NAD. 100 μL of the heated extract (containing NADH only), 25 μL of the unheated extract (containing NAD and NADH) and 50 µL of standards of known NADH (Roche) concentrations (ranging from 0.0625 to 1 µM) dissolved in carbonate-bicarbonate buffer were loaded in duplicate onto a 96-well microplate along with blanks (carbonate-bicarbonate buffer). Volumes were adjusted to 100 µL with carbonate-bicarbonate buffer and 150 μL of a reaction buffer was added into each well. Reaction buffer contained Bicine 133 mM, EDTA 5.33 mM, Methylthiazolyldiphenyl-tetrazolium bromide (MTT) 0.56 mM, Phenazine Ethosulfate (PES) 2.21 mM, Ethanol 0.67 M, and Alcohol Dehydrogenase (Sigma-Aldrich) 40 U/mL. The absorbance was followed spectrophotometrically at 560 nm every 15 s over a 15 min period (Safire 2; Tecan, Maennedorf, Switzerland). Blank values were subtracted from all samples and NAD amounts were calculated by subtracting NADH values from total NAD^+^ NADH concentrations. The protein content was measured using the remaining cell lysate using the BCA protein assay reagent kit (Pierce) according to the manufacturer’s instructions and used for normalization. Ratio of NADH/NAD^+^ values are expressed as percentage of control values (NADH/NAD^+^ of 6.49 +/− 0.65 with basal NADH cellular content of 205.15 ± 23.84 pmol/mg prot).

### Statistical analysis

A minimum of 20 neurons per culture were analyzed for each experiment. All data are presented as means ± SEM. Paired Student’s *t*-test or One-way ANOVA followed by *Dunnett’s post hoc test* have been used to determine statistical significance (*p < 0.05; **p < 0.01; ***p < 0.005).
